# Conceptualizing mental disorders as deviations from normative functioning

**DOI:** 10.1038/s41380-019-0441-1

**Published:** 2019-06-14

**Authors:** Andre F. Marquand, Seyed Mostafa Kia, Mariam Zabihi, Thomas Wolfers, Jan K. Buitelaar, Christian F. Beckmann

**Affiliations:** 1 Donders Centre for Cognitive Neuroimaging, Donders Institute for Brain, Cognition and Behaviour, Radboud University, Nijmegen, the Netherlands; 2 Department of Cognitive Neuroscience, Radboud University Medical Centre, Nijmegen, the Netherlands; 3 Department of Neuroimaging, Centre for Neuroimaging Sciences, Institute of Psychiatry, King’s College London, London, UK; 4 Karakter Child and Adolescent Psychiatric University Centre, Nijmegen, the Netherlands; 5 Oxford Centre for Functional Magnetic Resonance Imaging of the Brain (FMRIB), University of Oxford, Oxford, UK

**Keywords:** Neuroscience, Prognostic markers, Diagnostic markers

## Abstract

Normative models are a class of emerging statistical techniques useful for understanding the heterogeneous biology underlying psychiatric disorders at the level of the individual participant. Analogous to normative growth charts used in paediatric medicine for plotting child development in terms of height or weight as a function of age, normative models chart variation in clinical cohorts in terms of mappings between quantitative biological measures and clinically relevant variables. An emerging body of literature has demonstrated that such techniques are excellent tools for parsing the heterogeneity in clinical cohorts by providing statistical inferences at the level of the individual participant with respect to the normative range. Here, we provide a unifying review of the theory and application of normative modelling for understanding the biological and clinical heterogeneity underlying mental disorders. We first provide a statistically grounded yet non-technical overview of the conceptual underpinnings of normative modelling and propose a conceptual framework to link the many different methodological approaches that have been proposed for this purpose. We survey the literature employing these techniques, focusing principally on applications of normative modelling to quantitative neuroimaging-based biomarkers in psychiatry and, finally, we provide methodological considerations and recommendations to guide future applications of these techniques. We show that normative modelling provides a means by which the importance of modelling individual differences can be brought from theory to concrete data analysis procedures for understanding heterogeneous mental disorders and ultimately a promising route towards precision medicine in psychiatry.

In most areas of medicine, biomarkers that objectively indicate disease state have revolutionized diagnosis and treatment allocation. In contrast, psychiatric disorders are still diagnosed exclusively on the basis of symptoms and biological tests to assist diagnosis or treatment allocation remain to be developed [1]. This yields clinical groups that are highly heterogenous, both in terms of clinical presentation and underlying biology, which is a major barrier to understanding underlying mechanisms and developing better treatments [1–3]. This is widely recognized at a theoretical level and over the years, many different theoretical models have been proposed to explain the heterogeneity of psychiatric disorders [4–9]. These emphasize the myriad pathological mechanisms that may converge on the same symptoms in different participants [4, 5] and that the same underlying biological risk factors may result in a different clinical phenotype in different individuals depending on the context, genetic background and critical time window [4, 9]. Heterogeneity remains the dominant theme even in recent large-scale theoretical initiatives such as the Research Domain Criteria (RDoC) [6, 7] and the European Roadmap for Mental Health Research (ROAMER) [8]. All these theoretical models are founded on an implicit recognition of the importance of modelling individual differences within and across clinical cohorts [10, 11]. It is very important to recognize, however, that this broad theoretical recognition is not reflected in the data analysis strategies employed in practice. Instead, the overwhelming majority of analysis approaches remain focussed on group averages (e.g. the ‘average patient’) and regard individual differences principally as noise.^1^

Normative modelling is an emerging approach that can address this challenge by providing statistical inferences at the level of the individual with respect to an expected pattern [12]. This is analogous to the widespread use of normative growth charts in paediatric medicine to map child height or weight as a function of age with respect to centiles of variation in a reference population [13]. Normative modelling generalizes this notion by substituting these variables for clinically relevant variables then applying automated statistical techniques to map centiles of variation across the cohort. This is increasingly used to map variation between cognitive, clinical or demographic variables and quantitative biomarkers derived from neuroimaging [12, 14–17]. The key feature of normative modelling that makes it useful for stratifying cohorts is that it permits the detection and mapping of distinct patterns of abnormality in individuals without requiring a consistent neurobiological signature across all individuals.

Whilst early applications focused on brain development and ageing [16, 18], normative modelling has recently been shown to be highly promising for psychiatry [12, 14, 17, 19–21]. First, to map variation related to brain development and ageing in psychiatric disorders, which is appealing given the neurodevelopmental basis of mental disorders [22]. For example, neurodevelopmental normative models have been used in the context of schizophrenia [17, 19], attention deficit/hyperactivity disorder (ADHD) [20, 23] and autism [21, 24] to help understand the emergence of mental disorders as deviations from an expected developmental trajectory and identify individuals following an atypical trajectory. Second, normative modelling can also be abstracted beyond development to chart the spectrum of functioning across any cognitive domain. It has been used, for example, to chart variation in reward systems via mappings between trait measures of reward sensitivity and reward-related brain activity [12]. Finally, normative models can help to understand healthy variation and move beyond simple dimensional theories of mental disorders [25].

Here, we provide a unifying review of normative modelling for charting individual variation across different behavioural, demographic and biological dimensions, thereby helping to understand heterogeneity within clinical cohorts. We first provide a statistically grounded, yet non-technical overview of its conceptual underpinnings and propose a framework to link the many different methodological approaches that have been proposed. Second, we outline connections between normative modelling and existing approaches for tackling heterogeneity including clustering [26] and ‘brain age’ approaches that characterize subjects in terms of a difference between a brain-derived predicted age and true chronological age [27, 28]. Third, we survey the literature employing normative modelling in clinical conditions. Fourth, we discuss normative modelling as a tool for finding structure in large cohorts, which is important given the recent shifts towards ‘big data’ neuroscience and large population-based cohorts [29–31]. Finally, we provide recommendations for future studies and critically evaluate the limitations of normative modelling.

## Introduction to normative modelling for understanding heterogeneity in clinical cohorts

**Fig. 1:**
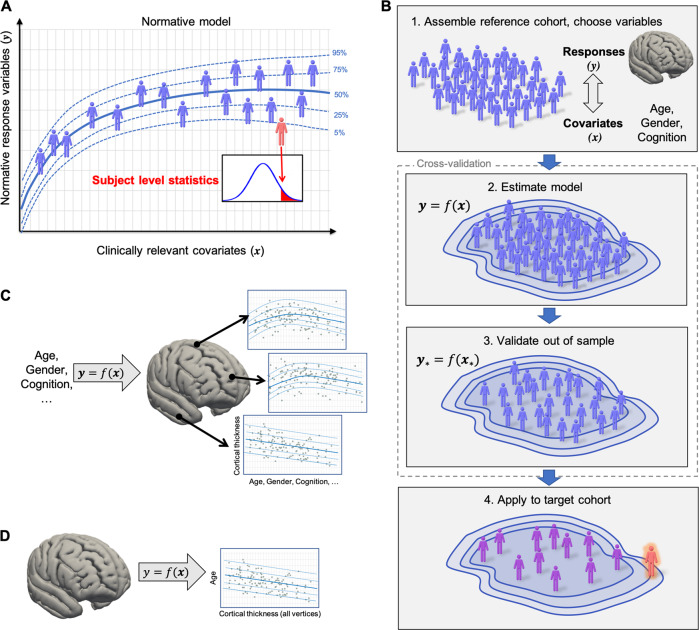
Conceptual overview of normative modelling. **a** Normative modelling is similar to the use of growth charts in paediatric medicine, except the conventional response variable (e.g. height or weight) is substituted for a quantitative biological readout (e.g. regional brain activity). The classical covariates (age and sex) can also be substituted for clinically relevant variables. Normative modelling provides statistical inference at the level of each subject with respect to the normative model (red figure). **b** Procedural overview of normative modelling. After the choice of reference cohort and variables, the normative model is estimated, before being validated out of sample on new response variables and covariates (y* and x*, respectively). Finally, the estimated model can be applied to a target cohort (e.g. clinical cohort). **c** A common configuration for normative modelling of neuroimaging data, where a separate normative model is estimated for each sampled brain location. This can be described by a set of functions (y = f(x)) predicting neurobiological response variables (y) from clinical covariates (x). **d** Normative models can also be estimated for the opposite mapping, where brain measures are chosen as covariates and age or other covariates are chosen as a response variable. See text for further details

Normative modelling is a statistical framework for mapping between behavioural, demographic or clinical characteristics and a quantitative biological measure, providing estimates of centiles of variation across the population (Fig. 1a). Normative modelling provides a concrete method for studying individual differences and parsing heterogeneity across cohorts because it provides statistical inferences at the level of the individual participant as to the degree to which each individual deviates from the normative pattern and allows these deviations to be mapped in each individual. In other words, normative modelling provides a way to quantify and characterize the manner in which different individuals deviate from the expected pattern, and from one another. Importantly, this does not require that atypicalities overlap across participants (e.g. in the same brain regions) or even that a consistent pattern of deviation exists. Therefore, this accommodates the convergence of multiple pathological pathways on the same symptoms in different individuals [4]. This is clearly different from case-control analyses, which all focus on first order statistics (group means), thereby seeking a consistent pattern of atypicality (i.e. the ‘average patient’). In a case-control context, heterogeneity becomes apparent via inflation of the model residuals and ultimately decreases sensitivity for detecting disorder-related effects. In contrast, normative modelling explicitly models heterogeneity because it focuses on modelling individual variation around the mean using second order statistics (variances). Therefore, normative modelling explicitly characterizes and quantifies the heterogeneity underlying clinical conditions at a finer grained level than is afforded by group averages. Normative modelling also does not require that the clinical group can be cleanly partitioned into subtypes [26] although it can be used to generate features for clustering. Normative modelling can be used to estimate many different kinds of mappings based on the variables chosen, but here we focus on mappings between behavioural or demographic measures and a quantitative biological readout, most commonly derived from neuroimaging.

Procedurally, normative modelling involves four steps (Fig. 1b): First, a reference cohort and a set of variables are chosen to define the mapping and population over which variation is measured. Second, a statistical model is estimated to model variance in a response variable (a.k.a. target or dependent variable) from a set of clinically relevant covariates (predictor or independent variables) across the reference cohort. For example, one may estimate a normative model for cortical thickness as a function of age and gender using a population-based reference cohort. Third, it is necessary to assess the accuracy of the normative model for predicting the response variable (e.g. mean-squared error, explained variance). To ensure accurate estimates of generalizability, this must be performed on withheld data (e.g. under cross-validation). Finally, this model can be applied to quantifying the deviations of samples from a target cohort (e.g. clinical cohort) with respect to this reference model.

Many regression models have been proposed for normative modelling, including hierarchical linear models, polynomial regression, quantile regression, support vector regression and Gaussian process regression (Table 1). The estimation of normative models is conceptually similar to classical growth charts, for which many approaches have been proposed [13]. In both cases, the data make multiple demands from the regression model including ensuring precise estimation of outer centiles (where data are sparsest), ensuring centiles vary smoothly as a function of the covariates (and do not cross) and the ability to estimate deviations for individual samples via analytical formulae (e.g. *Z*-scores) [32]. In order to provide a conceptual framework linking these approaches, we categorize different approaches according to three criteria: (i) the choice of covariates and response variables; (ii) the degree to which the model separates different sources of variation and (iii) the degree to which the model permits statistical inference the individual level. However, other features are also important, for instance ability to model non-linear relationships.

**Table 1:** Studies utilizing normative modelling techniques in clinical conditions

Clinical phenotype	Normative response variable	Covariates	Reference cohort	Target cohort	Algorithm	Separate variance components	Single subject prediction	Ref.
ADHD diagnosis	Functional connectivity measures derived from resting fMRI	Age	Population-based cohort	Participants with ADHD	Polynomial regression	No	Numerical	[20]
ADHD diagnosis	Brain volume	Age and gender	Healthy reference cohort	Adults with ADHD	Gaussian process regression	Yes	Statistical	[23]
ADHD symptoms	Reward-related brain activity derived from task fMRI	Delay discounting	Healthy volunteers	Healthy volunteers	Gaussian process regression and extreme value statistics	Yes	Statistical	[12]
Autism	Cortical Thickness	Age and gender	Typically developing cohort	Participants with autism	Gaussian process regression and extreme value statistics	Yes	Statistical	[24]
Autism	Cortical thickness	Age	Typically developing cohort	Participants with autism	Local polynomial regression	No	Statistical	[21]
Autism	Alpha band brain activity derived from electro-encephalogaphy	Age	Typically developing cohort	Participants with autism	Local polynomial regression	No	Numerical	[44]
Bipolar disorder	Brain volume	Age and gender	Healthy reference cohort	Adults with Bipolar disorder	Gaussian process regression	Yes	Statistical	[17]
Cognition: processing speed	Age	Brain volume	Healthy volunteers	Healthy volunteers	Support vector regression	No	Statistical	[14]
Cognition: Inhibitory Control	Task-related fMRI data	Age	Healthy volunteers	Healthy volunteers	Hierarchical linear modelling	No	Numerical	[45]
Sustained attention	Functional connectivity measures derived from resting fMRI	Age	Population-based cohort	Participants with ADHD	Polynomial regression	No	Statistical	[20]
Mild cognitive impairment and dementia	Brain volume	Age	Population-based cohort	Participants with mild cognitive impairment and Alzheimer’s disease	Partial least squares and quantile regression	No	Statistical	[34]
Mild cognitive impairment and dementia	Brain volume	Age, sex, total grey- and white matter volume, total cerebrospinal fluid, MRI field strength	Multi-study healthy reference cohort	Healthy participants, participants with mild cognitive impairment and dementia	Gaussian process regression	Yes	Statistical	[16]
Psychosis symptoms	Age	Cognitive scores measuring executive function, memory, complex cognition, social cognition and sensorimotor processing	Developmental study cohort containing typically developing adolescents and adolescents with psychosis spectrum symptoms (2 levels) and other psychopathologies	Developmental study cohort containing typically developing adolescents and adolescents with psychosis spectrum symptoms (2 levels) and other psychopathologies	Linear regression	No	Numerical	[19]
Schizophrenia	Brain volume	Age and gender	Healthy reference cohort	Adults with Schizophrenia	Gaussian process regression	Yes	Statistical	[17]
Schizophrenia	Longitudinal cortical thickness measures derived from structural MRI	Age	Healthy reference cohort	Children, adolescents and adults with childhood onset schizophrenia	Penalized spline models	No	Numerical	[46]

Different methods are classified in terms of the choice of covariates and response variables, whether they estimate separate variance components and in terms of the degree of single-subject prediction that they provide (see text for details)

## Choice of covariates and response variables

A simple way to categorize different approaches is in terms of the variables that define the mapping. One common configuration (Fig. 1c) uses age as a covariate, often in combination with other clinical or demographic variables to predict a quantitative biological readout. However, there are other possibilities: for example, the mapping can be inverted such that age is the response variable which is predicted from clincal or demographic variables (Fig. 1d). This is the approach used by ‘brain age’ models [27, 28] which use multivariate regression to predict age from a pattern of brain-derived measures. For linear models, it is obvious that an association can be detected in either direction simply by inverting the linear model. However, the interpretation of the centiles of variation and regression coefficients differs and we consider that charting variation over the biological readout is more appealing because it directly mirrors the use of growth-charting in paediatric medicine.

As noted, normative models are also not restricted to charting variation across development. By substituting age for other variables, normative models can chart variation in any kind of mapping, for example to link cognitive scores with brain activity patterns [12].

## Separating different sources of variation across the cohort

**Fig. 2:**
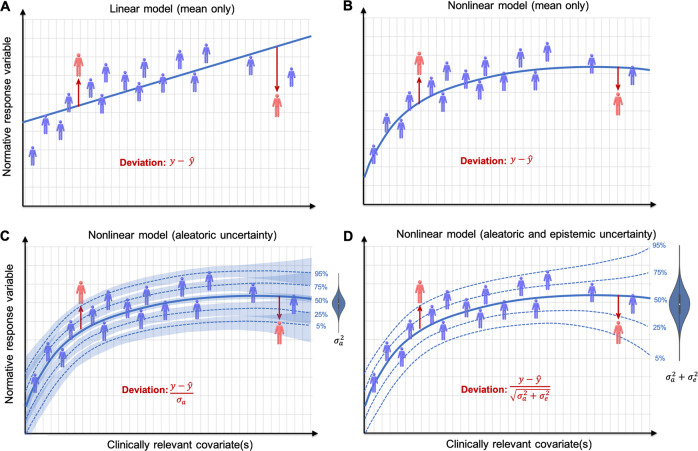
Separating different sources of uncertainty in normative modelling. Panels **a** and **b** show the simplest approach for normative models which do not quantify uncertainty at all (**a**: linear model, **b**: non-linear model). Instead, deviations from the model (red figures) are assessed via the residuals from a regression function (blue lines). In red, the corresponding equation for assessing deviations from the model is shown where deviation from the normative model are assessed simply as the difference between the true (*y*) and predicted ($\widehat y$) normative response variable for each subject. **c** Some models estimate centiles of variation explicitly either via separate model fits or post hoc to the initial regression fit (blue dotted lines). This captures ‘aleatoric’ or irreducible variation in the cohort which shows how subjects vary across the population ($\sigma _a^2$). However, there is also uncertainty associated with each of these centiles of variation (shaded blue regions), which is highest in regions of low data density and should be accounted for. **d** Some models separate and take all sources of variation into account (i.e. also including ‘epistemic’ uncertainty ($\sigma _e^2$), which can be reduced by the addition of more data). This allows the model to automatically adjust predictions, becoming more conservative in regions where data are sparse. This is shown by a widening of the statistical intervals, although note that these intervals now have a different interpretation to those in (**c**). For example, the right-most figure in (**d**) would not be judged as an outlier, whereas the same figure may be judged as an outlier in models that do not account for all sources of uncertainty (**c**). This is important to prevent a subject being declared as ‘atypical’ simply because of data sparsity. See text for further details

Normative modelling principally aims to model variation across the cohort over and above estimation of mean effects. To achieve this effectively, it is important to separate different sources of variation, most importantly to differentiate actual variation within the data (i.e. across participants) from variability due to parameter and model uncertainty (i.e. induced variability due to a lack of data). In normative modelling, we quantify these variabilities using two types of uncertainty commonly defined in machine learning [33]: (i) irreducible (or ‘aleatoric’) uncertainty that reflects true underlying variability that cannot be reduced with more data; (ii) reducible (or ‘epistemic’) variation that reflects parameter uncertainty or ignorance about the true model and can be reduced by more data. Aleatoric uncertainty is of primary interest for stratification because it reflects variation across subjects whereas epistemic uncertainty is nuisance variation that it is desirable to minimize. The degree to which different approaches account for these sources of variability can be classified hierarchically (Fig. 2): the simplest approach involves estimating the mean effect only and assessing deviations from the expected pattern using the model residuals (Fig. 2a, b) [19, 27]. Whilst this is appealing in its simplicity, it provides no estimate of variation across the cohort and cannot provide statistical inferences at the individual level (see below). This has been addressed in different ways, for example via estimating confidence intervals via a post hoc regression between the residuals of the model against the true response variable [14] or using quantile regression to directly estimate centiles of variation in the data [34]. These approaches provide statistical estimates of variation within the population and can indicate if a particular participant deviates from the expected pattern at a given confidence level, but they do not fully account for different sources of uncertainty, e.g. uncertainty in the estimation of the centiles (Fig. 2c). Bayesian methods such as Gaussian process regression [12, 16, 35] provide one solution to this problem (Fig. 2d) by estimating distinct variance components and providing predictions for each participant that account for all sources of uncertainty. This is important for two reasons: first, it provides estimates of centiles of variation within the reference cohort that are not influenced by data density; second, it allows all sources of uncertainty to be taken into account when making predictions. This provides the desirable property that inferences become more conservative in regions of the input space where data are sparse. With these complementary purposes in mind, it may be desirable to report different variance components separately.

## Degree of individual prediction

The ability of the model to perform single participant inference can be classified hierarchically. At the simplest level (‘numerical inference’; Fig. 2a, b), the model only provides numerical deviations (i.e. residuals of the target cohort from the reference model). This is the approach taken by most brain age approaches [27] and permits group-level inferences about how deviations correlate—for example—with symptoms. In contrast, some models provide estimates of centiles of variation within the population (‘statistical inference’; e.g. quantile regression [34] and Bayesian techniques [12, 16]). This provides inferences as to whether each individual deviates from the model at a given statistical significance level (Fig. 2c). Some studies have derived variance estimates via post hoc regression on the model residuals [14] and atypicality cutoffs could also be defined post hoc, although this must be done on unseen data to remain unbiased. However, as described above, it is important to recognize whether predictions account for all sources of variance. If they do not, they may yield overly optimistic inferences. This can be addressed using models that estimate separate variance components for different types of variance and account for all uncertainty in the predictions (Fig. 2d).

In addition to mapping deviations in individuals, it is often desirable to estimate participant-level summary statistics for the overall deviation from the normative pattern. Different methods have been proposed for this, e.g.: combining deviations across all voxels [16] or modelling the most extreme deviations in each subject using extreme value statistics [12].

## Relationships to other approaches for parsing heterogeneity

Normative modelling is complementary to the predominant approach for tackling heterogeneity in mental disorders, i.e. subtyping using clustering algorithms. Clustering has been widely applied [26] and is often useful. However, it also suffers from limitations: first, clustering assumes that the clinical group can be cleanly partitioned into subtypes. However, this assumption is seldom evaluated, which is problematic because clustering algorithms always yield a result, regardless of whether clusters are ‘really there’ [26, 36]. Second, clustering focuses on group averages and does not fully model individual variation within clusters. In other words, most clustering algorithms regard piece-wise constant clusters as atomic units. Whilst some algorithms provide ‘soft’ cluster assignments that capture some variation within clusters, this cannot accurately model, for example, a spectrum of functioning. In contrast, normative modelling shifts the analytical focus: (i) away from group means to understanding cohort variation (i.e. from first- to second-order statistics); (ii) towards understanding variation across individuals and (iii) towards mapping deviations at the level of individual.

Normative modelling is complementary to alternative techniques for individual prediction; for example, supervised discriminative models [3] can assess the degree of group separation in a case-control sense and therefore provide predictions that are specific for certain disorders. On the other hand, normative modelling can be used to understand the variation across the cohort independently of the clinical labels.

Brain age models are related and complementary to normative modelling. They can be considered as a type of normative model which estimate the opposite mapping (i.e. brain readouts as covariates and age as the response variable; Fig. 1d). As noted, choosing age as a covariate mirrors growth-charting in paediatric medicine and allows the regional deviations in each subject to be mapped, which is desirable for interpretation. In contrast, brain age models condense a complex multivariate pattern into a single number (a deviation between true and predicted age). This is often useful because it summarizes a complex pattern by an interpretable score. On the other hand, it provides limited ability to stratify individuals or identify which brain regions underlie any observed deviation. This is important because different subjects may have the same predicted brain age because of distinct underlying abnormality patterns [27].

## Applications of normative modelling in psychiatry

Normative modelling has been applied to many clinical phenotypes: unsurprisingly, many applications have focused on studying changes in brain organization across the lifespan. More recently, studies have emerged applying normative modelling to map the biological heterogeneity underlying mental disorders (Table 1).^2^ Taken together, the applications reviewed here show the flexibility of normative modelling for many different clinical phenotypes, on the basis of different clinical and biological measures. Moreover, they highlight the value of normative modelling for studying individual differences in that they show that: (i) a potentially small number of patients have alterations in the same brain regions and (ii) that the pattern of individualized regional differences detected by normative models can be very different to case-control differences. For example, normative deviations may be partially consistent with case control-effects [17], very different [21] or evident in the absence of case-control effects [24].

## Normative modelling for big data

Normative modelling is useful for understanding variation in ‘big data’ cohorts. This parallels an increasing focus in clinical neuroimaging towards acquiring large population-based cohorts that capture a wide range of clinically relevant variation [29–31]. The conventional motivation is to increase statistical power for the detection of subtle effects [37]. While this is undoubtedly important, such cohorts also provide an excellent opportunity to understand structure in heterogeneous clinical populations. Normative modelling is ideal for this due to its focus on understanding variation rather than detecting mean effects and can be used to find distinct and potentially non-overlapping patterns of abnormality.

On the other hand, big data cohorts introduce challenges, including computational scaling of analytical methods to many data points, requiring availability of modelled variables across all subjects and dealing with nuisance variation. For example, most large datasets include data from multiple study sites, which increases the risk of missing data and introduces the possibility that observed deviations could be related to site variance. To address these concerns, careful stratification procedures during model fitting and cross-validation and explicitly modelling different sources of variance are important (e.g. using hierarchical models). Alternatively, for predictions on new sites, normative models may be recalibrated on held-out normative control data to ensure that the normative model remains appropriate for the new data sample. There are many ways to achieve this [38], but a simple approach involves adjusting the mean, slope and variance after fitting a post hoc regression to withheld subjects.

Another use for normative models is for calibrating measures on different scales to a common normative reference. In other words, separate normative models can be estimated for different cognitive and biological mappings. This has the effect of rescaling different variables to a common reference range (for example, *Z*-statistics reflecting the number of standard deviations each subject is from the population norm). This forms an ideal set of features for the application of clustering algorithms, in the spirit of precision medicine. Relative to application to the raw data, this increases interpretability by scaling diverse data to population norms and also can tease apart correlated symptom domains more clearly than using clinical or biological data alone.

Ultimately, estimating normative models to link multiple phenotypic measurements with their multifaceted biological underpinnings is likely to be very important to: (i) understand disorders across multiple domains in the spirit of RDoC and ROAMER; (ii) identify different groups of patients with different atypical mechanisms; (iii) to better understand healthy variation and how this relates to the mechanisms of mental disorders and (iv) to move beyond simple dimensional theories of mental disorders [25].

## Study design considerations

The applications above show that normative modelling is very flexible given the choice of covariates, response variables, target cohort and reference cohort. The choice of reference cohort is particularly crucial; it is important that it captures a wide range of variation in the reference population. In paediatric medicine, the typical choice is a population-based cohort containing thousands of participants. In psychiatry, it is also common to estimate normative models using population-based cohorts that include participants across the full range of functioning (i.e. both healthy and with disorders) [19, 20], although this is not the only option and may not always be the optimal choice. For neuroimaging, several large population-based cohorts are being acquired [29, 31, 39], however these often focus on specific lifespan periods [29] and are frequently enriched for individuals ‘at-risk’ for mental disorders [31, 39]. Moreover, since these cohorts aim to address multiple questions, they may lack rich clinical phenotyping measures that are valuable for characterizing deviations (see below). Another option is to apply normative models to existing cohorts (e.g. based on case-control designs) [17, 23, 24]. In such cases, either the whole cohort or only the healthy participants can be used for the reference cohort. If the whole cohort is used, it is important to remember that under a case-control paradigm the frequency of the clinical phenotype is usually much higher than the population prevalence (e.g. equal numbers of cases and controls). If only the healthy subjects are used as the reference, this can be considered an approximation to a population-based cohort, which is reasonable if the prevalence of the clinical phenotype in the wider population is relatively low. In both cases, the deviations should be interpreted with respect to the cohort chosen. Regarding the choice of the target cohort, this can be the same as the reference cohort, provided the predictions are derived in an unbiased manner, for example, under cross-validation.

## Limitations

Normative modelling is a bottom-up approach to map variation and should not be considered a substitute for hypothesis testing or top-down theory driven approaches. Rather, it is desirable to combine the benefits of both. For example, combining top-down theory driven approaches with supervised discriminative models [3] and normative models. These can be used, respectively, to assess the degree of group separation in a case-control sense and to map the variation across individuals with respect to the theory-driven model.

Another important consideration is that normative models do not directly indicate whether the deviations obtained are clinically relevant. For example, deviations may be biologically meaningful yet unrelated to psychopathology, or they may be a result of artefactual variation (e.g. site variation). Therefore, external validation of derived deviations on external measures such as symptoms, genotype and environmental factors is crucial, as are careful data preprocessing and checking procedures.

## Future developments: towards precision psychiatry

Normative modelling provides a method to map deviations from an expected pattern at the individual level. This has been helpful to understand individual variation in the context of brain development or ageing and also for mapping variation across multiple cognitive domains and measures of brain organization. This provides the potential to understand individual variation across a multi-dimensional cognitive space, consistent with initiatives such as RDoC [6–8]. However, work remains to be done as to how these can be integrated and to determine the clinical relevance of individual variability. In other words, how, when and why individual variability turns into vulnerability or resilience. Indeed, a high priority is to develop methods that can convert deviations from normative models to a stratification of individuals. As noted, training clustering algorithms on the deviations from multiple normative models is one option [12, 26]. However, conventional ‘hard’ clustering algorithms allocate each subject to a single cluster and do not accommodate the possibility of multiple overlapping mechanisms operating in different individuals. Therefore, a more promising route may be using ‘soft’ clustering algorithms or other latent variable models that allow subjects to be allocated to multiple potentially overlapping clusters or risk profiles [40]. Another area of future work involves explicitly modelling spatial information, which may increase sensitivity for detection of spatially distributed patterns of abnormality [41–43].

## Conclusions and outlook

We have surveyed the emerging literature employing normative modelling to mental disorders. We have shown that these methods are highly flexible: they can naturally applied to estimating centiles of variation in brain growth and to mappings between many aspects of behaviour, cognition and biology. The most important feature of normative models is their ability to make predictions at the level of individual with respect to a normative pattern and that they shift emphasis from studying mean effects to understanding individual variation. They provide a means by which modelling individual differences can be brought from theory to concrete data analysis procedures and therefore a promising route towards precision medicine in psychiatry.

## Footnotes

1 This includes classical analysis methods based on the general linear model (e.g. *t*-tests and analysis of (co-) variance) plus classical and supervised machine learning classification models (e.g. linear discriminant analysis, logistic regression and support vector machines) and unsupervised clustering methods (e.g. k-means), where—at best—confound regressors are used to remove some of the cross-subject variation.

2 Note that we include some studies that can also be considered brain age studies but that share salient features with normative models (e.g., ref. [14]), we refer the reader elsewhere for more detailed reviews of brain age studies [27, 28].

## References

[1] Kapur S, Phillips AG, Insel TR (2012). Why has it taken so long for biological psychiatry to develop clinical tests and what to do about it?. Mol Psychiatry.

[2] Scarr E, Millan MJ, Bahn S, Bertolino A, Turck CW, Kapur S (2015). Biomarkers for Psychiatry: The Journey from Fantasy to Fact, a Report of the 2013 CINP Think Tank. Int J Neuropsychoph.

[3] Wolfers T, Buitelaar JK, Beckmann C, Franke B, Marquand AF (2015). From estimating activation locality to predicting disorder: a review of pattern recognition for neuroimaging-based psychiatric diagnostics. Neurosci Biobehav Rev.

[4] Cicchetti D, Rogosch FA (1996). Equifinality and multifinality in developmental psychopathology. Dev Psychopathol.

[5] Cannon TD, Keller MC (2006). Endophenotypes in the genetic analyses of mental disorders. Annu Rev Clin Psychol.

[6] Insel T, Cuthbert B, Garvey M, Heinssen R, Pine DS, Quinn K (2010). Research Domain Criteria (RDoC): toward a new classification framework for research on mental disorders. Am J Psychiatry.

[7] Insel TR, Cuthbert BN (2015). Brain disorders? Precisely. Science.

[8] Schumann G, Binder EB, Holte A, de Kloet ER, Oedegaard KJ, Robbins TW (2014). Stratified medicine for mental disorders. Eur Neuropsychopharmacol.

[9] Marin O (2016). Developmental timing and critical windows for the treatment of psychiatric disorders. Nat Med.

[10] Foulkes L, Blakemore SJ (2018). Studying individual differences in human adolescent brain development. Nat Neurosci.

[11] Seghier ML, Price C (2018). Interpreting and utilising intersubject variability in brain function. Trends Cogn Sci.

[12] Marquand AF, Rezek I, Buitelaar J, Beckmann CF (2016). Understanding heterogeneity in clinical cohorts using normative models: beyond case-control studies. Biol Psychiatry.

[13] Cole TJ (2012). The development of growth references and growth charts. Ann Hum Biol.

[14] Erus G, Battapady H, Satterthwaite TD, Hakonarson H, Gur RE, Davatzikos C (2015). Imaging patterns of brain development and their relationship to cognition. Cereb Cortex.

[15] Eavani H, Hsieh MK, An Y, Erus G, Beason-Held L, Resnick S (2016). Capturing heterogeneous group differences using mixture-of-experts: application to a study of aging. Neuroimage.

[16] Ziegler G, Ridgway GR, Dahnke R, Gaser C, Alzheimer’s Dis N (2014). Individualized Gaussian process-based prediction and detection of local and global gray matter abnormalities in elderly subjects. Neuroimage.

[17] Wolfers T, Doan NT, Kaufmann T, Alnæs D, Moberget T, Agartz I (2018). Mapping the heterogeneous phenotype of schizophrenia and bipolar disorder using normative models. JAMA Psychiatry.

[18] Brewer JB (2009). Fully-automated volumetric MRI with normative ranges: translation to clinical practice. Behav Neurol.

[19] Gur RC, Calkins ME, Satterthwaite TD, Ruparel K, Bilker WB, Moore TM (2014). Neurocognitive growth charting in psychosis spectrum youths. JAMA Psychiatry.

[20] Kessler D, Angstadt M, Sripada C (2016). Growth charting of brain connectivity networks and the identification of attention impairment in youth. JAMA Psychiatry.

[21] Bethlehem R, Seidlitz J, Romero-Garcia R, Lombardo M. Using normative age modelling to isolate subsets of individuals with autism expressing highly age-atypical cortical thickness features. BioRxiv 2018:1–23.

[22] Insel TR (2014). Mental disorders in childhood shifting the focus from behavioral symptoms to neurodevelopmental trajectories. JAMA.

[23] Wolfers T, Beckman CF, Hoogman M, Buitelaar JK, Franke B, Marquand A. Individual differences v. the average patient: mapping the heterogeneity in ADHD using normative models. Psychol Med. 2019;311:1727–8. 10.1017/S0033291719000084 PMC7083555 30782224

[24] Zabihi M, Oldehinkel M, Wolfers T, Frouin V, Goyard D, Loth E, et al. Dissecting the heterogeneous cortical anatomy of autism spectrum disorder using normative models. Biol Psychiatry Cogn Neurosci Neuroimaging. 2018. 10.1016/j.bpsc.2018.11.013 PMC6551348 30799285

[25] Holmes AJ, Patrick LM (2018). The myth of optimality in clinical neuroscience. Trends Cogn Sci.

[26] Marquand AF, Wolfers T, Mennes M, Buitelaar J, Beckmann CF (2016). Beyond lumping and splitting: a review of computational approaches for stratifying psychiatric disorders. Biol Psychiatry: Cogn Neurosci.

[27] Cole JH, Franke K (2017). Predicting Age Using Neuroimaging: Innovative Brain Ageing Biomarkers. Trends Neurosci.

[28] Cole JH, Marioni RE, Harris SE, Deary IJ (2019). Brain age and other bodily ‘ages’: implications for neuropsychiatry. Mol Psychiatry.

[29] Miller KL, Alfaro-Almagro F, Bangerter NK, Thomas DL, Yacoub E, Xu JQ (2016). Multimodal population brain imaging in the UK Biobank prospective epidemiological study. Nat Neurosci.

[30] Van Essen DC, Smith SM, Barch DM, Behrens TEJ, Yacoub E, Ugurbil K (2013). The WU-Minn Human Connectome Project: an overview. Neuroimage.

[31] Volkow Nora D., Koob George F., Croyle Robert T., Bianchi Diana W., Gordon Joshua A., Koroshetz Walter J., Pérez-Stable Eliseo J., Riley William T., Bloch Michele H., Conway Kevin, Deeds Bethany G., Dowling Gayathri J., Grant Steven, Howlett Katia D., Matochik John A., Morgan Glen D., Murray Margaret M., Noronha Antonio, Spong Catherine Y., Wargo Eric M., Warren Kenneth R., Weiss Susan R.B. (2018). The conception of the ABCD study: From substance use to a broad NIH collaboration. Developmental Cognitive Neuroscience.

[32] Borghi E, de Onis M, Garza C, Van den Broeek J, Frongillo EA, Grummer-Strawn L (2006). Construction of the World Health Organization child growth standards: selection of methods for attained growth curves. Stat Med.

[33] Gal Y (2016). Uncertainty in deep learning.

[34] Huizinga W, Poot D, Vernooij M, Rothschupkin G, Ikram M, Rueckert D (2018). A spatio-temporal reference model of the aging brain. Neuroimage.

[35] Rezek I, Beckmann C. Models of disease spectra. arXiv:1207.4674 [stat.ML]: arXiv preprint, 2012.

[36] Liu Y, Hayes DN, Nobel A, Marron JS (2008). Statistical significance of clustering for high-dimension, low-sample size data. J Am Stat Assoc.

[37] Smith SM, Nichols TE (2018). Statistical challenges in “big data” human neuroimaging. Neuron.

[38] Sansom PG, Ferro CAT, Stephenson DB, Goddard L, Mason SJ (2016). Best practices for postprocessing ensemble climate forecasts. Part I: selecting appropriate recalibration methods. J Clim.

[39] Calkins ME, Merikangas KR, Moore TM, Burstein M, Behr MA, Satterthwaite TD (2015). The Philadelphia Neurodevelopmental Cohort: constructing a deep phenotyping collaborative. J Child Psychol Psychiatry.

[40] Ruiz FJR, Valera I, Blanco C, Perez-Cruz F (2014). Bayesian nonparametric comorbidity analysis of psychiatric disorders. J Mach Learn Res.

[41] Kia SM, Marquand A. Normative modelling of neuroimaging data using scalable multi-task gaussian processes. ArXiv. 2018;1–12.

[42] Kia SM, Beckmann CF, Marquand AF. Scalable multi-task Gaussian process tensor regression for normative modeling of structured variation in neuroimaging data. ArXiv. 2018;1808.00036.

[43] Kia SM, Marquand A. Neural processes mixed-effect models for deep normative modeling of clinical neuroimaging data. arXiv. 2018;1812.04998.

[44] Lefebvre A, Delorme R, Delanoe C, Amsellem F, Beggiato A, Germanaud D, et al. Alpha waves as a neuromarker of autism spectrum disorder: the challenge of reproducibility and heterogeneity. Front Neurosci. 2018;12:662. 10.3389/fnins.2018.00662 PMC6174243 30327586

[45] Ordaz SJ, Foran W, Velanova K, Luna B (2013). Longitudinal growth curves of brain function underlying inhibitory control through adolescence. J Neurosci.

[46] Alexander-Bloch AF, Reiss PT, Rapoport J, McAdams H, Giedd JN, Bullmore ET (2014). Abnormal cortical growth in schizophrenia targets normative modules of synchronized development. Biol Psychiatry.

