# Correction: Conceptualizing mental disorders as deviations from normative functioning

**DOI:** 10.1038/s41380-019-0451-z

**Published:** 2019-06-26

**Authors:** Andre F. Marquand, Seyed Mostafa Kia, Mariam Zabihi, Thomas Wolfers, Jan K. Buitelaar, Christian F. Beckmann

**Affiliations:** 1 Donders Centre for Cognitive Neuroimaging, Donders Institute for Brain, Cognition and Behaviour, Radboud University, Nijmegen, the Netherlands; 2 Department of Cognitive Neuroscience, Radboud University Medical Centre, Nijmegen, the Netherlands; 3 Department of Neuroimaging, Centre for Neuroimaging Sciences, Institute of Psychiatry, King’s College London, London, UK; 4 Karakter Child and Adolescent Psychiatric University Centre, Nijmegen, the Netherlands; 5 Oxford Centre for Functional Magnetic Resonance Imaging of the Brain (FMRIB), University of Oxford, Oxford, UK

**Correction to: Molecular Psychiatry**

10.1038/s41380-019-0441-1;

published online 14 June 2019

In the original version of this article, panel labels in the legend of figure 1 were incorrectly positioned alongside non-corresponding panel descriptions. The legend of figure 1 has now been updated. This has been corrected in both the PDF and HTML versions of the article.

